# Novel Molecular Insights about Lactobacillar Sortase-Dependent Piliation

**DOI:** 10.3390/ijms18071551

**Published:** 2017-07-18

**Authors:** Ingemar von Ossowski

**Affiliations:** Department of Veterinary Biosciences, Faculty of Veterinary Medicine, University of Helsinki, Helsinki FIN-00014, Finland; ingemar.von.ossowski@helsinki.fi; Tel.: +358-(0)-2941-57180

**Keywords:** sortase-dependent pili, pilin, Gram-positive, *Lactobacillus rhamnosus*, *Lactobacillus ruminis*, commensal, niche-adaptation factor, adhesion

## Abstract

One of the more conspicuous structural features that punctuate the outer cell surface of certain bacterial Gram-positive genera and species is the sortase-dependent pilus. As these adhesive and variable-length protrusions jut outward from the cell, they provide a physically expedient and useful means for the initial contact between a bacterium and its ecological milieu. The sortase-dependent pilus displays an elongated macromolecular architecture consisting of two to three types of monomeric protein subunits (pilins), each with their own specific function and location, and that are joined together covalently by the transpeptidyl activity of a pilus-specific C-type sortase enzyme. Sortase-dependent pili were first detected among the Gram-positive pathogens and subsequently categorized as an essential virulence factor for host colonization and tissue invasion by these harmful bacteria. However, the sortase-dependent pilus was rebranded as also a niche-adaptation factor after it was revealed that “friendly” Gram-positive commensals exhibit the same kind of pilus structures, which includes two contrasting gut-adapted species from the *Lactobacillus* genus, allochthonous *Lactobacillus rhamnosus* and autochthonous *Lactobacillus ruminis*. This review will highlight and discuss what has been learned from the latest research carried out and published on these lactobacillar pilus types.

## 1. Introduction

For humans, the extended arm and hand is a useful tool that lets us navigate certain social settings, often as a friendly feeling gesture epitomized by a welcoming handshake, but then sometimes as a protective-aggressive response that comes by wielding a clenched fist. In a mechanistic sense for certain microscopic bacteria, they operate in their surrounding conditions somewhat similarly through the use of long proteinaceous limb-like protrusions that reach out from the periphery of the cell wall envelope. These multi-subunit appendages are commonly known as pili (sing., pilus) or fimbriae (sing., fimbria) and can be detected in both a number of Gram-negative and Gram-positive genera and species, with each type having an archetypal polymeric structure held together by either non-covalent or covalent forces, respectively (for detailed review, see [1,2]). As a function, surface pili mediate the opening contact between the bacterial cell and its physical environment, this accomplished by virtue of their variable lengths and then by being imparted with a characteristic adhesiveness for targeted substrates. Historically, the Gram-negative pili were discovered about sixty years ago [3], and subsequent to extensive characterization, they have come to be known by their complexity, both from a functional and structural perspective (for detailed review, see [4]). On the other hand, it was about fifteen years later when pilus-like formations were first recognized among Gram-positive pathogens [5,6], and as these surface features play an essential and early role during the invasive colonization of various body regions, they were soon deemed a virulence factor of such damaging bacteria.

Gram-negative pili come in different forms and depending on which kind (i.e., chaperone-usher, curli, type IV, and the type III and IV secretion), they have diameters and lengths within the ranges of 4–14 nm and 1–20 µm, respectively [4]. Typically, they have characteristic quaternary macromolecular structures that can appear as a helical rod-like assemblage of several different pilin-called protein subunits [4]. By comparison, the architectural arrangement of the Gram-positive pilus is more simplified than its counterpart in Gram-negative bacteria, and although two varieties can be distinguished by electron microscopy (EM), one short and the other long (reaching upwards of 0.5 or 3 µm), a good deal of recent scientific interest has been drawn to the lengthier pilus structures [2]. Typically, these pili are comprised of just two to three types of pilin subunits, each with its own distinct role and location in the pilus structure. For this, a transpeptidase enzyme called the pilus-specific C-type sortase catalyzes the head-to-tail assembly of the pilins into the final polymerized form, which in most instances has the thickness of a single subunit molecule (~2–3 nm) [7] and lacks a complex quaternary organization [8]. Surface piliation such as this is generally described as being “sortase-dependent”, the occurrence of which can be found only amongst the Gram-positive bacteria. Largely stemming from their prospective use as a vaccine candidate against disease-causing Gram-positive bacteria [9], the ensuing years have seen much effort to characterize the function and structure of sortase-dependent pili from various differently piliated pathogens. As a consequence, many molecular aspects about the pathogen-derived pilus types in Gram-positive bacteria have already emerged and continue to do so. However, the universal moniker as a trait for virulence no longer seemed to apply when, more recently, it had been revealed that sortase-dependent pili could assemble themselves on the cell surface of non-pathogenic bacteria. Here, it was visually shown that the gut-friendly commensals *Lactobacillus rhamnosus* [10,11,12,13], *Lactobacillus ruminis* [14], and *Bifidobacterium bifidum* [15] are also surface-piliated natively, at which point the sortase-dependent pilus began to carry the new name of a niche-adaptation factor.

This present review will concentrate on the newly found lactobacillar pilus types, whereupon an attempt is made to chronicle some of their molecular characteristics and properties that have been reported in the literature during the past few years. For this, an emphasis is placed on what new information has been learned concerning the biology of sortase-dependent pili, and as well on how these macromolecular surface features can contribute to gut colonization behavior. 

## 2. Sortase-Dependent Pilus: An Overview

As mentioned above, the various sortase-dependent pili that originate from Gram-positive pathogens have already become well characterized in many respects, these including their genetic organization, assembly and structural composition, and as well molecular and cellular functionality. In this section, a description will be given on what is now understood as an overall consensus about these pathogen-derived pili after about a dozen years of intensive research and study. For more in-depth information on this specific topic the reader is referred to several recent reviews (e.g., [2,16,17,18,19]).

### 2.1. Gene Expression

**Figure 1:**
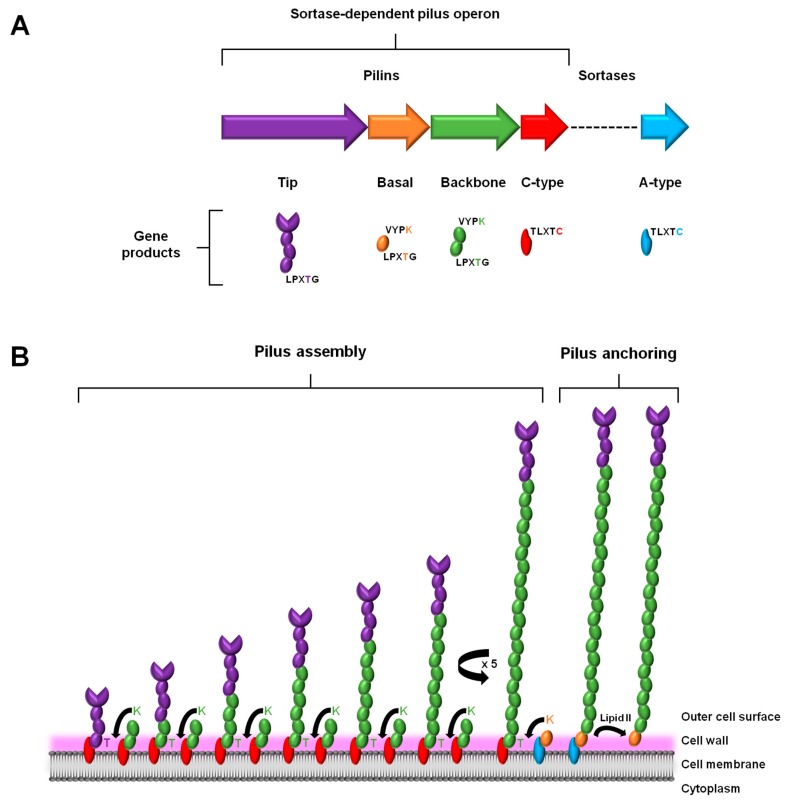
Genetics and mechanism behind the pilin assembly and cell wall anchoring of sortase-dependent pili. (**A**) Depicted is a schematic representation of a characteristic sortase-dependent pilus operon, which includes the genes for the tip, basal, and backbone pilin subunits and the pilus-specific C-type sortase enzyme. The housekeeping A-type sortase gene is invariably found elsewhere along the genome. Examples of the various pilin gene products are illustrated, and for each of these pilins, the location of the N-terminally located pilin motif (VYPK) and the LPXTG motif at the C-terminus are indicated correspondingly. Sortase gene products are shown with the conserved catalytic motif (TLXTC) in the vicinity of the active-site cleft. Key residues of each amino acid motif are highlighted by color. (**B**) Schematically depicted from left to right are the sequential steps leading up to the assembly and anchoring of a typical three-subunit sortase-dependent pilus. See (**A**) for the identities of the various pilins and sortases. (Refer to main text for details).

As a characteristic genome feature of the piliated Gram-positive pathogens, the genes that encode the proteins for assembling a sortase-dependent pilus are clustered close together in a genetic island or operon [20]. Such a tandem arrangement of genes ensures the harmonized expression of the protein products that are involved with constructing a macromolecular pilus structure (Figure 1A). This genomic attribute is rather simply organized and includes the genes for the different pilin subunits, the one major type that comprises the polymerized length of the pilus backbone, and those deemed minor or ancillary that are positioned at the base and tip of the pilus. Also found within this gene cluster is the locus for a pilus-specific C-type sortase enzyme. However, some exceptions to this description also exist, e.g., where in the genes for the basal pilin-proteins are missing (e.g., [21,22]) and with additional sortase genes being present (e.g., [23,24]). Moreover, depending on the particular Gram-positive genus and species, the genome can encode a fimbrial operon for more than just one type of pilus. As an example of this, certain species of *Streptococcus* [23] and *Corynebacterium* [24] are known to contain the operon loci for expressing three structurally distinct pilus types.

For the most part, the basic genetic principles that underlie the regulation of sortase-dependent pilus expression are not yet fully deciphered and understood, although some studies are now beginning to reveal more about the mechanisms and factors involved with these controlling processes. While in many instances the cellular production of pili can be constitutive, one interesting phenomenon that has been noticed is the heterogeneity in pilus expression for a number of piliated Gram-positive pathogens, such as *Enterococcus faecalis* [25,26], *Streptococcus pyogenes* [27], *Streptococcus pneumoniae* [28], *Streptococcus gallolyticus* [22], *Corynebacterium pilosum* [29], and *Corynebacterium renale* [29], in which case not all of the cells are observed to display surface piliation. Additional studies indicate that for this bistable pattern of pilus expression there can also exist a mixed population of highly and lowly piliated cells [30,31,32]. Gene expression from the sortase-dependent pilus operons is controlled mainly at the transcriptional level, with the regulatory loci and elements being located in upstream regions and the regulatory proteins ordinarily acting as an activator [33]. For instance, the manifestation of bistability as found with *S. pneumoniae* PI-1 pili involves the RlrA positive regulator that not only activates the expression of the corresponding pilus operon [32], but as well, uses a positive feedback mechanism to regulate its own production [34]. Interestingly, an entirely different regulatory feature was described to account for the heterogeneous pilus expression of *S. gallolyticus*, in which the control of its *pil1* fimbrial operon includes a phase variation mechanism that combines the length modification of an upstream leader peptide with ribosome-mediated transcriptional attenuation [35]. As a reason for the lack of homogeneity in the piliated cell population, it has been proposed to represent an adaptive survival strategy that allows bacterial cells to not only adhere and colonize the host environment via their pili, but, by being either non-piliated or less piliated, also able to evade those defenses of the host immune system that are directed against surface piliation [16,36].

### 2.2. Assembly and Anchoring

In a manner consistent with its namesake, the assembly and anchoring of the sortase-dependent pilus entails the covalent cross-linking action by two transpeptidase sortases: (1) the pilus-specific C-type for initiating and polymerizing together the various pilin-proteins and (2) the housekeeping A-type for attaching the fully assembled structure to the cell wall (for detailed review, see [37,38,39]) (Figure 1). As the site of pilus assembly is restricted to the cell surface, the various protein components are exported through the cell membrane via the Sec pathway [40]. This also necessitates that each of the sortases and the pilin subunits contain hydrophobic stretches to enable their membrane attachment, but also so that the bulk of each protein is within the cell wall region. For the two types of sortases, this occurs through transmembrane domains in their N-terminal regions [41,42]. On the other hand, for the different pilin subunits (tip, basal, and backbone), and similarly for various sortase-dependent surface proteins, they possess a characteristic C-terminal region that contains hydrophobic aliphatic amino acids and a tail end of positively charged lysine and/or arginine residues. In addition, extended out from this stretch of membrane-bound protein there are five amino acids (LPXTG) that serve as an important recognition and cleavage motif for both sortase enzymes [40]. Normally, when the sortases catalyze a break in this pentapeptide region, it occurs at the bond between the threonine and glycine residues. To accomplish this, each sortase enzyme has a large open cleft that contains an active site cysteine, which itself is part of a conserved catalytic motif (TLXTC) and during cleavage will form a thioacyl intermediate with the threonine of the LPXTG motif [43].

A prototypical model for describing sortase-dependent pilus assembly originates from a study of the surface piliation in *Corynebacterium diphtheriae* [24] (Figure 1B). To initiate the pilus assembly process, two C-type sortases, one coupled to a tip pilin and another carrying a backbone pilin, will first connect together their accompanying protein subunits [37]. Here, the lysine (K) of the so-called pilin motif (WXXXVXVYPKN) near the N-terminal of the backbone pilin and the LPXTG-motif threonine in the tip pilin become cross-linked via a covalent isopeptide bond, such that the pilin subunits are orientated head-to-tail. Following this, and done in the same manner, a succession of backbone pilins is added sequentially to the lengthening pilus structure. After a sufficient number of protein subunits have been incorporated, pilus biogenesis undergoes termination, which, depending on the pilus type, can be facilitated in one of two ways. For those pili with an archetypal three-subunit structure that also includes another ancillary pilin (in addition to the one at the pilus tip), pilus assembly proceeds to terminate via the presence of a housekeeping A-type sortase carrying the basal pilin, with C-type sortase-catalyzed K-T isopeptide bond formation then following and linking this particular subunit to the last backbone pilin of the pilus structure [44,45]. While now attached to the A-type sortase, a covalent bond forms between the basal pilin of the assembled pilus and the peptidoglycan layer, a process involving the lipid II moiety and leading to cell wall anchoring. For the pilus types not having the basal pilin, the housekeeping sortase simply catalyzes the same series of reactions as above, but instead with a backbone-pilin subunit, although less optimally [46]. Intriguingly, while the basal pilin is proposed as a likely candidate for signaling the end to pilus assembly [47], what then actually triggers this termination process in its absence is less clear and still remains to be established.

Much of the work to identify and confirm the relative positioning of the various subunit types within an assembled sortase-dependent pilus was done using EM in combination with immunogold labeling of pilin-proteins (i.e., immuno-EM) [24]. This technique served especially well, and derived from the findings representative structural models could be postulated, with each having in common the predicted location of the tip, basal, and backbone pilin subunits. However, as a peculiarity, the ancillary basal pilin was in some instances also detected along the length of the polymerized pilus backbone [24,48,49]. Because the primary structure of many basal subunits often tend to also exhibit what resembles a canonical pilin motif, a likely reason for its additional location in the pilus is that it was merely a random occurrence and involves being mistakenly recognized and incorporated by the C-type sortase. Since VYPK-like pilin motifs are seldom found in tip pilins, the critical lysine needed for K-T isopeptide bond formation would be missing, which then precludes any possible insertion of the tip subunit between adjacent backbone pilins. Alternatively, it was proposed that another amino acid motif of YXLXETXAPXGY (the so-called E-box) near the C-terminal region is involved in mediating the decorative outer attachment of basal pilins along the pilus backbone [50]. Although subsequent to this, it was reported that the E-box glutamate primarily plays a part in maintaining the proper fold of the pilin-proteins [51], and as well in the formation of intramolecular isopeptide bonds [52,53]. Yet another study using the more advanced cryo-EM technique appeared to refute the notion that the ancillary subunits are attached to the pilus in an atypical manner [8]. Here, it was established that for the *S. pneumoniae* TIGR4 pilus its structure consists of a repeating length of RrgB major pilins, with the RrgA and RrgC minor pilins confined to only the pilus tip and base, respectively. Significantly, this study also highlighted the limits of interpreting results obtained from conventional immuno-EM when undertaking to structurally characterize sortase-dependent pili, as there exists the tendency for antibody-related artifacts to sometimes occur [8].

### 2.3. Structural Composition

Apart from the tip, basal, and backbone pilins having a number of basic features in common, such as signaling regions at the N- and C-termini and certain consensus motifs, it is notable as well that their corresponding tertiary structures consist of protein domains with similar folds. Three-dimensional (3D) structures of these pilin subunits are comprised of the CnaA and CnaB domains (for detailed review, see [17,18]), both of which represent variant folds of the immunoglobulin (Ig)-like domain that was originally observed in the staphylococcal collagen adhesin (Cna) protein [54,55]. The structural fold of the CnaA and CnaB domains is dominated by a core of nine and seven β-sheets, respectively [56], which, depending on the pilin type, also contains characteristic regions of loops and α-helices, but as well, extra β-strands and other domains. Intriguingly, Gram-positive pilins are distinguished by their spontaneous formation of intramolecular (or internal) isopeptide bonds, with these occurring either between lysine and aspartate (K-D) or lysine and asparagine (K-N), and thus unlike the intermolecular K-T isopeptide bond that holds together different pilin subunits [52,53]. While perceived as a substitute for disulfide bridges [57], this intramolecular covalent bonding is formed autocatalytically, in which the spontaneity of the reactions involved is set forth when the CnaA and CnaB domains are folded together such that each creates a hydrophobic environment and wherein the key residues are positioned in optimal geometric proximity [7]. Moreover, here it seems that a particular residue environment is tailored to either K-D or K-N isopeptide bond formation [7].

**Figure 2:**
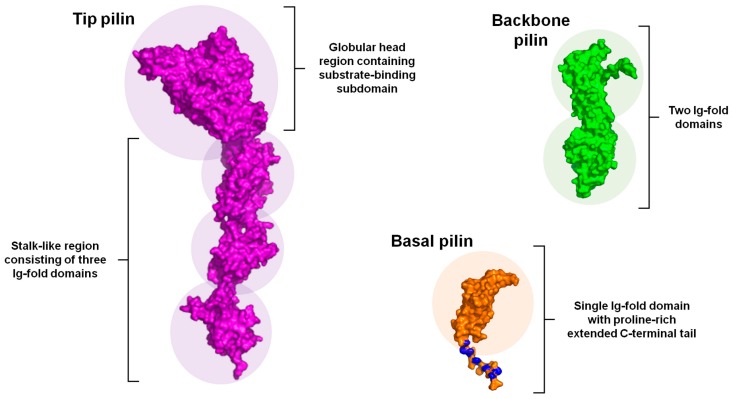
Three-dimensional view of the tip, backbone, and basal pilin structures within a typical sortase-dependent pilus. Shown are representative tertiary structures of the three types of pilin subunits. The structure of the tip pilin is comprised of a stalk-like region consisting of three Ig-fold domains (e.g., CnaA or CnaB), upon which rests a globular head region that contains the substrate-binding domain. The C-terminal LPXTG peptide is normally situated within the bottom subdomain. The backbone pilin structure is shown having two Ig-fold domains, with the linking lysine and the LPXTG peptide located within the upper and lower domains, respectively. The basal pilin structure is depicted with a single Ig-fold domain that includes a proline-rich (blue) extended C-terminal tail. All domain regions are highlighted by circular background shading. Structural visualization of the tip, backbone, and basal pilins was rendered by PyMol (http://www.pymol.org/) using protein atomic coordinates (2WW8, 3B2M, and 3LKQ, respectively) retrieved from the RCSB Protein Data Bank (http:// http://www.rcsb.org/pdb/home/home.do).

To date, representative crystal structures of the tip, basal, and backbone pilin subunits have been solved from a variety of pilus types and pathogen sources, and with these results it is observed they have various structural features in common, but as well, those that distinguish them well-apart from one another (Figure 2). For instance, the tip pilin is by far the largest subunit in the sortase-dependent pilus, typically consisting of four separate subdomains, three of which having the CnaA/CnaB folds and that together resemble a stalk-like structure upon which is situated a larger fourth domain [58,59,60,61]. In contrast to the two Ig-like domains, this fourth domain is globular and contains recognized-binding subdomains, and it is these characteristics that make the tip pilins suitable for adhesive interactions. On the other hand, the basal pilins are the smallest in size of the pilus proteins and normally encompass one to three CnaB domains, which themselves may or may not display intramolecular isopeptide bond formation [62,63,64]. Of some interest, a universal structural attribute of the basal pilins is the presence of a C-terminal extension rich in hydrophobic proline residues. Because a similar tail-end region is not found in the tip and backbone pilins, its function in the basal subunit might be related to the events leading up to the covalent attachment of the pilus to the outer cell surface [63]. Typically for the backbone pilins, their structural makeup can be double- to quadruple-domained, and as well including both CnaA and CnaB folds [18]. In most cases, intramolecular isopeptide bonds are produced in these domains, which are assumed important in providing the structural rigidity and stability necessary for withstanding the severe shearing conditions encountered by sortase-dependent pili in natural environs [57]. A key feature of backbone (and basal) pilins is the so-called “linking lysine” that forms an isopeptidyl connection with the LPXTG-motif threonine of a nearby adjacent subunit. More often than not, this lysine is within the VYPK peptide region (pilin motif), but invariably this particular residue is found in the “head” N-terminal domain somewhere near the C-terminus [18]. Comparatively, the N-terminal domain of the backbone pilin has the most flexible structure, as its isopeptide bond tends to form slowly or then only during pilus assembly [65]. It is for this reason that when backbone pilins are produced recombinantly, the N-terminal domain will readily succumb to proteases. Here, it is thought that the structurally supple nature of the N-terminal domain will make the lysine residue more accessible for docking to an adjoining pilin via the C-terminal threonine, but as well, then allow for sealing and merging at the head-to-tail interface of two backbone subunits as they become polymerized into a pilus fiber [66].

### 2.4. Functional Attributes

From a physical perspective, piliated Gram-positive pathogens are considered more competitive than their non-piliated counterparts by being able to adhere to available target sites from a distance. This capacity for first contact with the host is due to the exceptional length of pilus structures, but as well, to the exploitation of their characteristic adhesiveness. For instance, numerous studies have shown that the virulence of piliated Gram-positive pathogens can stem from pilus-mediated adhesion, and it has become widely recognized that the tip pilins are the main adhesive component of pili. Primary structure examination of tip pilins has revealed that several contain sequence for a von Willebrand adhesin (VWA) domain, e.g., such as in *C. diphtheriae* SpaC [20], *E. faecalis* EbpA [67], *S. pneumoniae* RrgA [58], *S. agalactiae* PilA [68], and *S. pyogenes* AP-1 [69]. The VWA domain can be found in many other bacterial proteins, but as well, various archaeal and eukaryotic proteins, and as a functional property it is known to interact with the epithelial ECM [70]. Accordingly, an adherence to ECM substrates, such as collagen, fibronectin, fibrinogen, and laminin, has been attributed to various Gram-positive pathogen-sourced pili and tip subunits [22,71,72,73]. Moreover, for some piliated pathogens (e.g., *C. diphtheriae*, *S. pneumoniae*, and *S. pyogenes* [74,75,76]) their adhesion to epithelial cells is also pilus mediated. As host cell invasiveness by most pathogens first requires an attachment to the epithelium, and in particular to any exposed ECM proteins, those genera and species with such substrate-adhesive pili are functionally specialized for this purpose and have a distinct advantage for successful colonization and dissemination.

Then again, while most functional analyses of pathogen-derived sortase-dependent pili point to ECM components as adhesion targets, a recent study had reported that intestinal mucus can also act as a binding substrate [77,78]. Here, the Pil3 pilus of the opportunistic pathogen *S. gallolyticus* was shown to mediate adherence to colonic mucus and was required for the colonization of the murine distal colon, all primarily through the action of its tip pilin (Pil3A). This offers the possibility that the pili of Gram-positive pathogens can as well promote bacterial colonization of the gut mucosa. In addition, sortase-dependent pili have an important role in the development of pathogenic biofilm communities, with a good example being those oral species of *Streptococcus* and *Actinomyces* involved with dental plaques and caries [79,80,81]. For these, the adhesive pilus contributes to the early stages of biofilm growth by participating in the primary attachment of bacterial cells to saliva-coated tooth surfaces. Further, as various types of infections are caused by enterococcal and streptococcal pathogens, their pilus-driven accumulation as biofilm (e.g., [22,26,68,69,75]) can often lead up to hindering or delaying wound healing [82]. As a final point, other evidence has begun to emerge that suggests the sortase-dependent pili of pathogenic Gram-positives behave much like many other surface proteins and are targeted by the host innate immune system, e.g., as demonstrated by their ability to elicit certain inflammatory [28,83] and phagocytic [84,85,86] responses. 

## 3. Sortase-Dependent Pili in Non-Pathogenic Lactobacilli

### 3.1. History of Discovery

After the discoveries that some pathogenic Gram-positive bacteria can be piliated on their outer surface, it was not until 2009 when sortase-dependent pili were also found present in the less harmful commensals. As it happens, there were actually two publications that year, both giving an account of surface piliation in *Lactobacillus rhamnosus* GG, a gut-adapted strain whose commercial reputation is that of a much advocated and used probiotic (for detailed review, see [87]). In June, a Belgian group published a study [13], in which, by using EM and negative staining, they observed emanating from the cell poles of an extracellular polysaccharide-deficient mutant of *L. rhamnosus* GG the presence of what was discerned to be pilus-like formations. Later on in October, another report was published by a multinational consortium of scientists that then confirmed these surface structures on *L. rhamnosus* GG cells as being sortase-dependent pili [11]. Findings for this were from a study that was largely a genomic comparison between the GG and LC705 strains of *L. rhamnosus*. Here, it was found that the *L. rhamnosus* GG genome contains loci for two different sortase-dependent fimbrial operons (so-called *spaCBA* and *spaFED*), each of which encoding for the predicted tip (SpaC and SpaF), basal (SpaB and SpaE), and backbone (SpaA and SpaD) pilin-proteins, and as well a C-type sortase enzyme (SrtC1 and SrtC2). On the other hand, the genome of the *L. rhamnosus* LC705 dairy starter strain was observed to contain the genes for only the *spaFED* pilus operon. Moreover, an examination of the deduced primary structure from the corresponding pilus operon genes had confirmed that each of the SpaCBA and SpaFED pilins contains the characteristic conserved sequence motifs and domains of a typical Gram-positive pilin subunit (see Table 1). Incidentally, the “Spa” prefix given to the names of the *L. rhamnosus* pili was adapted from the nomenclature used for designating each of the pilus types in *C. diphtheriae* and this corresponds to sortase-mediated pilus assembly [88].

Typically, the tried-and-tested means for determining the surface localization of sortase-dependent pili in Gram-positive bacteria has included the electrophoretic immunoblotting technique and the previously mentioned use of immuno-EM, with both necessitating the need of anti-pilin serum [89]. For the immunoblotting analysis of cells, an observed ladder-like pattern with compressed high-molecular-weight (HMW) protein bands corresponds to the various fragmented lengths of accumulated pilus fibers, and this is normally taken to indicate the presence and production of sortase-dependent pili. In the Kankainen et al. (2009) study, the above methods with SpaC pilin antiserum were used to establish the active expression of the *L. rhamnosus* GG *spaCBA*-encoded loci, and ultimately the assemblage of the produced pilin-proteins into what is now normally known as the SpaCBA pilus [11]. At this point, the results from the immuno-EM experiments had indicated the existence of the SpaC pilin subunit along the entire length of the SpaCBA pilus structure [11], and with its apparent predicted location at the pilus tip only confirmed afterward in a follow-up study some three years later [90].

Significantly though, a number of “firsts” were established for sortase-dependent piliation from the study by Kankainen and colleagues. These included the first descriptions of a piliated Gram-positive non-pathogen, a piliated *Lactobacillus* species, and functionally of a sortase-dependent pilus having an intestinal mucus-binding capacity [11]. For this latter finding, adherence to human intestinal mucus was revealed for the SpaC subunit itself and then as well in the context of the SpaCBA pilus, which was shown by blocking its binding ability through the use of anti-SpaC serum and a s*paC*-inactivated mutant of *L. rhamnosus* GG [11]. It is on such a basis that the authors of this study had concluded the mucoadhesive SpaC pilus protein is an important host colonization determinant in *L. rhamnosus* GG and likely a main contributing factor in the more pronounced persistence of this gut transient (allochthonous) strain. At the time, the unearthing of mucus-binding surface piliation in *L. rhamnosus* GG had represented an entirely new concept, and as a novel molecular mechanism it ushered in a new impetus to examine and understand what physiological and functional roles might be played in intestinal microecology and probiosis. Curiously as well, not only did these research findings draw some attention and interest within the related scientific community [91], but there also was an element of sensational newspaper and social media coverage aimed at the general public. 

### 3.2. Other Lactobacillar Pilus Operons

Following close on the heels of the above-mentioned findings came the expected further scrutiny of other *Lactobacillus* species, and from this it appeared that the genetic potential for sortase-dependent surface piliation is not too prevalent among other members of this genus. For instance, only the phylogenetically close “casei group” of lactobacilli (*Lactobacillus casei*, *Lactobacillus paracasei*, and *L. rhamnosus*) are containing the representative fimbrial *spaCBA* and *spaFED* operons [92,93,94], and where expectedly a reasonably high degree of sequence identity exists among the primary structures of the various predicted proteins at a species and strain level. Added to this, genomic evidence was brought forth that the sortase-dependent pilus genes are present in *Lactobacillus ruminis*, a gut-indigenous flagellated species taxonomically distant from the members of the casei group [95]. Here, it was found that the fimbrial operon (eventually named as *lrpCBA*; [14]) encodes a canonical set of three pilus proteins and single sortase that are distinguishable from those associated with the SpaCBA and SpaFED pili, thus appearing to represent another type of lactobacillar pilus. It was later confirmed in a published report that *L. ruminis* is a natively surface-piliated bacterium [14]. Further, based on a study that carried out in silico sequence predictions to estimate the genomic presence of sortase-dependent pilus operons, it was inferred from the data output that other potentially piliated lactobacilli are distributed in clades containing the *Lactobacillus composti*, *Lactobacillus brevis*, and *Lactobacillus parabrevis* species [96]. However, as of yet, any number of these pilus-producing lactobacilli still remains to be corroborated experimentally in further studies.

## 4. SpaCBA Piliation

Thus far among the three types of sortase-dependent pili in lactobacilli, the SpaCBA pilus from *L. rhamnosus* GG has become the best studied, which in large part is due to it being the first one discovered and made known to be produced by cells, but as well, stemming from a strong commercial interest in the science behind the advocated probiosis of its host strain. Up to now, any other published reports indicating the active expression of the s*paCBA* operon are limited to a just a few *L. rhamnosus* strains [10,12], but otherwise nearly all molecular and functional characterization studies have involved the *L. rhamnosus* GG SpaCBA pilus.

### 4.1. Genetics

As introduced beforehand, the genomic distribution of the *spaCBA* operon (i.e., *spaC*-*spaB*-*spaA*-*srtC1*) appears to be restricted to only the *L. casei*, *L. paracasei*, and *L. rhamnosus* species of the so-called casei group [12,92,93,94]. However, while so far the *spaCBA* loci are a near-common attribute of the *L. casei* and *L. paracasei* genomes, surface localization of the SpaCBA pilus is still to be established in these two species. On the other hand, the presence of the fimbrial *spaCBA* operon in *L. rhamnosus* seems to be proportionally more widespread in gut-sourced strains than those originating from dairy products [10,12]. This suggests that whereas a strong evolutionary pressure had prevailed to maintain mucoadhesive SpaCBA pili in the *L. rhamnosus* strains whose ecological niche is the mucosal gut, it was not sufficiently manifested for those strains isolated from a milk-based environment, since presumably an adaptive advantage would not have been similarly gained by this molecular trait [10,12]. Still, although mucus itself might be perceived as a unique selection determinant for acquiring the SpaCBA pilus-related loci, it is not a very dominant driving force, as there are also intestinal strains without the *spaCBA* operon [10,12]. Moreover, the *spaCBA* genes seem not to be overly prevalent in any *L. rhamnosus* strains that originate from other mucus-lined regions in the body, such as the vaginal cavity and the respiratory airways, including the mouth [10,12]. Further, because a pan-genomic appraisal of *L. rhamnosus* found that the *spaCBA* operon is not part of the essential core genome, and instead is included among the dispensable accessory loci, the SpaCBA pilus genes can be regarded as a genomic rarity in this species [12]. Thus, as it is clear the *spaCBA* operon is a genetic element not shared by all *L. rhamnosus* strains, this might suggest that the corresponding genes had evolved recently in the genome, potentially through the lateral transfer of sortase-dependent pilus loci from the closely related *L. casei* and *L. paracasei* species, or perhaps even more ancestrally from piliated gut bacteria like *Enterococcus faecium* and *Enterococcus faecalis* [12]. While purely speculative, the premise for such a lateral gene acquisition is biologically conceivable, given that a transposon-like insertion sequence (IS) is identifiable within a region at both flanking ends of the *L. rhamnosus spaCBA* operon [97], and as this type of genomic feature is known for helping expedite the gene movement process.

To date, much of what is known regarding the regulation of SpaCBA pilus production is based on a comparative in silico analysis of DNA segments bordering the *spaCBA* operon in the genome of the *L. rhamnosus* GG strain. In the study by Douillard and colleagues, a possible regulatory region that might serve to control constitutive expression of the *spaCBA* loci was found within a specific DNA sequence immediately upstream from the *spaC* gene, and which may have arisen in the genome as an *iso*-IS*30* element [97]. Here, putative -10 and -35 promoter DNA elements were identified and include the pentameric 5′-TTGAA-3′ and hexameric 5′-TGGTCT-3′ sequences, respectively. Interestingly, in spite of their divergence from canonical consensus promoter sequences for RNA polymerase binding (i.e., -10 TATAAT and -35 TTGACA), the involvement of an alternative sigma (σ) factor was not being promoted as a possibility. However, in another study [98], it was found that the *spaCBA* operon from the *L. rhamnosus* E800 strain, which also produces SpaCBA pili [12], might instead be regulated by a promoter region that more closely resembles a typical -10 and -35 consensus sequence (i.e., TATAAT and TTGTTA, respectively), and thus better suited to being recognized by a standard σ^70^ factor. Moreover, similarly matching -10 and -35 recognition sites were also detected in the genome of *L. rhamnosus* GG, and as well for the LMS2-1 strain, though whose SpaCBA pilus production is not yet proven. Expectedly, these promoter sequences were not at all obvious for the *spaCBA* genes of the non-SpaCBA-piliated *L. casei* BL23 strain.

Owing to the juxtaposed presence of IS elements at both ends of the *L. rhamnosus* GG *spaCBA* operon, and therein the chance for increased genomic instability, Douillard and colleagues had also conducted an intriguing investigation to ascertain what impact evolutionary forces have on the permanency of the *spaCBA* loci in the genome [99]. In an examination of *L. rhamnosus* GG cells that had been propagated for at least 1000 generations under normal or highly stressed environmental conditions, the authors found that for the former there was no related gene loss from the genome, but for the latter when bile salt was used as the stressing agent, the IS elements became activated and appeared to cause some genetic recombination involving the *spaCBA* operon. In the context of the SpaCBA pilus as an evolving molecular trait, these findings clearly revealed the genomic plasticity of the *L. rhamnosus* GG strain and its adaptive prowess for change when confronted by a different environment.

### 4.2. Structure

**Figure 3:**
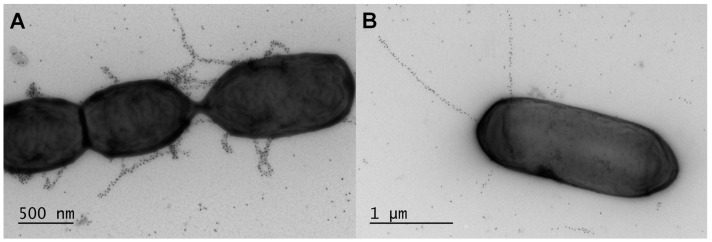
Visualization of native sortase-dependent piliation in lactobacilli by immuno-electron microscopy. Immunogold pilin-protein labeling and electron microscopy (EM) of bacterial cells were done using established techniques [89]. Long protruding structures representing the SpaCBA pili in *L. rhamnosus* GG (**A**) and LrpCBA pili in *L. ruminis* ATCC 25644 (**B**) are identified by immuno-labeling first with polyclonal antibody specific for the backbone-pilin subunit (SpaA and LrpA, respectively) and then protein A-conjugated gold particles (10 nm in diameter; black dots), followed by negative staining and electron microscopy (EM). Scale bars for each electron micrograph image are found at the bottom-left corner.

In terms of overall subunit architecture, lactobacillar SpaCBA piliation adopts the typical structure and molecular arrangement of a sortase-dependent pilus (Figure 3), wherein each of the three types of pilin-proteins is found situated in its predicted location. For instance, immuno-EM evidence from the Reunanen et al. (2012) study indicates that for the SpaCBA pilus, a repeating number of major SpaA pilins (~30 kDa) are polymerized linearly into the backbone structure, with the ancillary SpaB (~20 kDa) and SpaC (~90 kDa) subunits located at the anchored base and outwardly tip sites, respectively [90]. However, because a conventional EM technique was used to pinpoint the positioning of the individual pilin subunits, the experiments also tended to show the sporadic presence of the SpaB and SpaC pilins along the pilus structure. Notwithstanding the possibility that the added location for the ancillary pilins may well represent an artifact due to the use of polyclonal antiserum [8], this other site was nonetheless included into the schematic model being proposed for the SpaCBA pilus [90]. Here, the basal SpaB subunit is sandwiched between two SpaA pilins and as well appears on the pilus exterior, whereas the SpaC tip pilin only has the outer decorative location along the pilus backbone. However, as the mucoadhesive SpaC subunit is considered a key molecular determinant for gut colonization by *L. rhamnosus* GG, its extra presence would give SpaCBA pili a distinctively heightened ability to extend binding to the intestinal mucosa. Yet, in the years following this particular investigation there has been no further data or research to substantiate the presumptive structural uniqueness of the lactobacillar SpaCBA pilus, and in hindsight its suggested architectural divergence from an accepted norm of other sortase-dependent pili [8] might have been based on circumstantial observations and at the time simply a practical interpretation. Thus, in retrospect, it is quite plausible that in the case of SpaC, since the stalk-like domain regions of the tip pilin-protein structure could bear a topological resemblance to the backbone subunit, a certain portion of antibodies in the polyclonal serum raised against SpaC might as well have an affinity for SpaA pilins when they coalesce to form the structural backbone of the pilus. Alternatively, and more significantly, the recently solved crystal structure of *L. rhamnosus* GG SpaA did not reveal recognizable structural topologies for a possible mechanism that would lead to a peripheral attachment of SpaC along the pilus backbone [100]. Even so, in the context of competing for the binding sites available throughout the intestinal mucosa, any *L. rhamnosus* GG cells having SpaCBA pili with just a single SpaC adhesin per fiber would presumably retain the capacity to outlast other strains not piliated in a similar way.

As it stands, there have been no characterization studies examining the molecular aspects of how the various SpaCBA pilins are polymerized together into a pilus structure, although it is largely assumed that this will conform to the pilin assembly process established for other types of sortase-dependent pili. However, there is one related report in which the authors had tried to uncover what controlling role sortase specificity has on the assembly and anchoring of the *L. rhamnosus* GG SpaCBA pilus [101]. Previously, with another piliated Gram-positive host [102], it was revealed that a structural difference existing between the pilus-specific C-type and housekeeping A-type sortases is responsible for regulating their respective activity and specificity on the LPXTG peptide of different pilin subunits. For this, the C-type sortase has an extra N-terminal region that folds into a flexible helical loop (a so-called lid), wherein certain conserved residues mimic themselves as a pseudo-substrate that can interact with and cover the active site. While it was found that eliminating the lid from the sortase does not impede catalysis, the shifting or movement of the lid from the open cleft that contains the active site is viewed necessary for making the LPXTG-substrate accessible to the catalytic pocket. Interestingly, the authors of this particular study went on to suggest that the pilin-specificity of C-type sortases is not just dependent on recognizing the limited stretch of residues in the LPXTG motif, and instead most probably involves other molecular determinants [102]. Much along these lines, the study by Douillard et al. (2014) had found that by extending the amino acid sequence of the LPXTG motif at the C-terminus, this would allow differentiation between those substrates being recognized by a C-type or A-type sortase [101]. Presumably for the specificity of the SrtC1 and SrtC2 C-type sortases, among the backbone (SpaA and SpaD) and tip (SpaC and SpaF) pilins of *L. rhamnosus* GG, there occurs a conserved Gly-Xaa-Gly pattern proximately downstream of the LPXTG residues that is not present in the *L. rhamnosus* GG basal pilins (SpaB and SpaE) and various other sortase-dependent surface proteins. Having been designated the “triple glycine motif”, it was revealed their residue-substitution in the SpaA and SpaC pilins effects the normal specificity of the SrtC1 sortase, with a perturbed SpaCBA pilus polymerization as the observed outcome. With the added presence of non-polar glycines, the rationale is that the backbone of this lengthier peptide region (LPXTGGXG) would then have more conformational freedom and increased flexibility, and thus better catering to the structural nuances of C-type sortase specificity and binding affinity. Further, as a conserved version of this new motif was also identified in the backbone and tip pilins of other piliated Gram-positive genera and species [101], it might well be considered a universally shared recognition site of pilus-specific C-type sortases. Alternatively proposed for the housekeeping A-type sortase, whose structure lacks the lid feature, its specificity for LPXTG-like proteins does not require the existence of a triple glycine motif [101].

So far, among the three different SpaCBA pilins, the backbone SpaA subunit from *L. rhamnosus* GG has been crystallized [103] and its tertiary structure solved by X-ray crystallography [100]. As the first crystal structure of a pilin-protein derived from a non-pathogenic source, SpaA was shown to be structurally representative of other Gram-positive pilins, e.g., by both being multi-domained and adopting Ig-like folds. More specifically, the SpaA structure is made up of two CnaB subdomains, each identified with an internal isopeptide bond and an E-box motif [100]. However, the chemistry of the isopeptide bonds is of particular interest, since whereas this covalent bond in the N-terminal domain is typical and formed between lysine and asparagine residues (K-N), in the C-terminal domain region, the bond formation is uncharacteristic by involving an aspartate instead (K-D) [100]. It is worth mentioning that from the structural work on SpaA, this marked the first time that the crystal structure for the N-terminal domain of a Gram-positive pilin was determined, since generating good X-ray-diffracting crystals has normally been quite difficult due to the intrinsic flexibility of this protein region. Concerning the functional role of the E-box, despite showing that residue-substitution has a negative impact on the proteolytic and thermal stability of SpaA pilin-protein, polymerization of the SpaCBA pilus was unaffected and remained normal [100]. Interestingly, because in the study by Chaurasia and colleagues a structural likeness to a native-like pilus was found in the molecular packing of SpaA crystals, this led the authors to propose a macromolecular model of the SpaCBA pilus that shows the way in which the SpaA pilins are arranged together. Here it was observed that a head-to-tail orientation of SpaA proteins places the C-terminal LPXTG peptide region of each subunit in the vicinity of an open cavity next to the N-terminal pilin motif residues of the adjoining subunit. With the added contribution of an inter-domain hinge angle of 152° in each SpaA pilin, the polymerized repeats of SpaA subunits can be modeled as having an extended spiraled staircase shape, which should give both rigidity and flexibility to the pilus backbone structure, and thereby explaining how the SpaCBA pili are designed to withstand the harshness of the highly dynamic intestinal environment [100].

### 4.3. Adhesion

As previously mentioned [11], the SpaCBA pilus is inherently mucoadhesive, with the large-sized SpaC pilin being viewed as the main determinant for such binding behavior. This was further supported by the results of mucus-binding experiments performed with a recombinant SpaCBA-piliated lactococcal clone that had the SpaC subunit deleted from the pilus structure [104]. Interestingly, as also found with some pathogen-derived tip pilins, the primary structure of SpaC shows the presence of a VWA domain, wherein additionally there is included a short region that is partially homologous to the domain for a fucose-binding lectin [11]. Based on this, it is speculated that substrate binding by SpaC might be attributed to a lectin-type recognition mechanism, and then particularly since one of the prime constituents of mucus is glycosylated mucin protein [11]. This type of SpaC-glycan interaction is also supported by a recent study that indicates SpaC pilin can bind to the carbohydrate chains of mucin, with an essential role being played by the β-galactoside moiety at the non-reducing end [105].

In addition to having an ability to bind mucus, SpaCBA pili appear to mediate the adherence of *L. rhamnosus* GG to epithelial cells. For this, two different studies using mutated strains of *L. rhamnosus* GG, a *spaCBA* knockout mutant [106] and an isogenic *spaC* mutant [107], were able to show that the SpaCBA pilus, and explicitly the SpaC pilin [107], have a functional role in bacterial adhesion to the human intestinal Caco-2 cell line. Moreover, while a dedicated domain for binding ECM collagen cannot be detected within the SpaC primary structure, there is one report that indicates a binding interaction between SpaC and collagen can occur [108]. Here, it was found that the SpaC-collagen interaction is also subject to rapid dissociation and from this it had been speculated that this could allow for a quick detachment and attachment between the SpaCBA pilus and the host environment, such as in those disrupted regions of the gut epithelium where ECM proteins are exposed and available for binding.

While the 3D structure of SpaC is yet to be solved, its crystallization as a near full-length protein has been recently reported [109], and arising from this work were some interesting observations about the molecular determinants for substrate binding by this pilin subunit. For instance, conserved domain prediction via InterPro had revealed the VWA domain is located within the N-terminal region of the SpaC protein, whereas the domain for the CnaB-type fold is localized at the C-terminal end. Moreover, it was further revealed that SpaC contains the motif for a metal ion-dependent adhesion site (MIDAS; D-x-S-x-S T D), and for which many VWA domains use to bind metal ions [70]. Further, based on predictive homology comparison with the RrgA and GBS104 tip pilins, the MIDAS-containing VWA domain can be found at the tip and surrounded by two inserted arms, and functionally it has been implicated with collagen binding.

In a trio of published studies [108,110,111], atomic force microscopy (AFM) was used to examine and characterize in detail the subunit organization and binding mechanics of SpaCBA pili, and here some notable findings were revealed. For instance, in the first study it was observed that the SpaCBA pili can appear bundled together as a single structural unit, which was thought to originate from glycan-lectin or hydrophobic interactions involving the SpaC subunit [110]. This was further expanded upon by the second study where it was found that SpaCBA pili have a propensity for self-adhesion that is driven by homophilic interactions between SpaC pilins, and which potentially also contributes to bacterial cell aggregation [108]. Rather interestingly, when *L. rhamnosus* GG cells were experimentally subjected to being pulled apart, and depending on the amount of force used, the observed mechanical retort of the pili could be categorized in two possible ways: (1) as a zipper-like mechanism involving SpaC-SpaC binding at low applied force and (2) as a molecular nanospring at higher forces, in which pili have a springiness and thus able to withstand a sustained stress or shock. Finally, a third study went further to reveal that the mechanical forces behind SpaCBA pilus-mediated adhesion can vary with the type of target-binding substrate [111]. Here, the coiling and extension behavior (nanospring) of SpaCBA pili are what help mediate adhesion to solid surfaces, as was observed with hydrophobic or glycan (i.e., mucin) substrates. Oddly, it was found that the binding of *L. rhamnosus* GG cells to an intestinal epithelial cell line (Caco-2) was less dependent on the nanospring properties of the SpaCBA pilus, but instead had relied on a surface tethering between the corresponding cellular membranes (i.e., membranous nanotethers). Although laboratory-cultured Caco-2 cells lack mucus on their outer surface, it was hypothesized that the mechanical forces promoting *L. rhamnosus* GG adhesion to the gut mucosal epithelium will likely involve both the SpaCBA pilus nanosprings and the membranous nanotethers [111].

Interestingly from another study [112], it emerged that the recombinant form of the basal SpaB pilin-protein has adhesiveness for intestinal mucus, and for which the binding is about seven-fold more pronounced than that by SpaC. The peculiarity of this finding is that the SpaB primary structure shares no homology to any of the known (or otherwise) mucus-binding domains, and thus the ability of this basal pilin to adhere strongly to mucus substrates was unpredicted and rather surprising. By comparison, the backbone SpaA subunit also lacks any homology matches to recognizable mucus adhesion domains, but more predictably it showed no such affinity for mucus [112]. However, in the case of SpaB, its mucoadhesiveness seems not to rely on certain residues of a dedicated binding domain, but rather it might be explainable through a less specific type of adhesion mechanism. Stemming from the observation that SpaB has a higher isoelectric point than the SpaC and SpaA pilins (pI 8 vs. ~5), evidence was presented to show that the positively charged SpaB pilin might use electrostatic contacts to interact with negatively charged mucus glycans [112]. Still, while such a mucosal interaction was revealed for the recombinant SpaB protein, any such contribution to the affinity of the SpaCBA pilus for adherence to intestinal mucus could not be established, either through the use of SpaB-specific antiserum on *L. rhamnosus* GG cells [112], or then by using a recombinant lactococcal clone that produces SpaB-deleted SpaCBA pili [104]. Consequently, in terms of actual function, the purpose for which the basal SpaB subunit is strongly mucoadhesive remains rather puzzling and intriguing.

**Figure 4:**
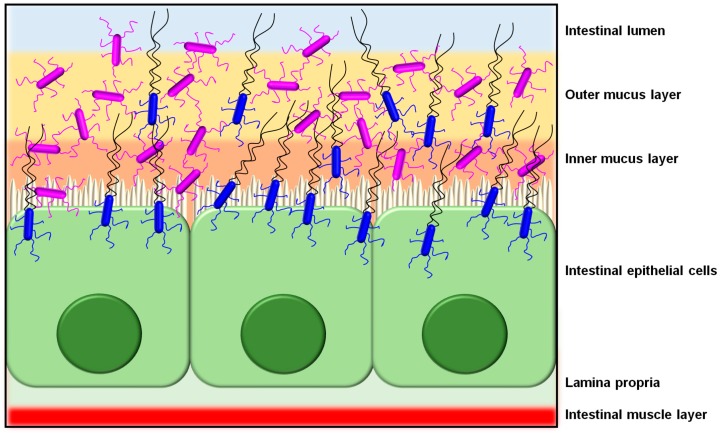
Schematic depiction showing the presumptive binding of piliated lactobacilli to the intestinal mucosa epithelium. Based on substrate-binding capacities, *L. rhamnosus* cells (purple) with their mucoadhesive SpaCBA pili would be predominantly found within the outer and inner mucus layers, whereas flagellated *L. ruminis* cells (blue) would be able to maneuver through this mucosal barrier and then, via their ECM-binding LrpCBA pili, attach themselves to the single cell layer of intestinal epithelial tissue. This cartoon image is representative only, and includes various aspects and features that are not drawn to scale.

As a host colonization strategy, the overall adhesive properties offered by SpaCBA piliation likely enables *L. rhamnosus* GG cells to extend their transient occupation of the intestinal niche (Figure 4), or at least more so than those strains without such pili [11]. Presumably, not only would the SpaCBA pilus facilitate the initial cell attachment to host binding sites, but once fastened, it also acts like the sortase-dependent pili from certain other Gram-positive bacteria by helping *L. rhamnosus* GG undergo self-aggregation to form biofilm, which could further enhance its colonization of the gut. For instance, from the analysis of knockout phenotypes for the *spaCBA* operon [106], and as well from the behavior of recombinant SpaCBA-piliated lactococci [14], SpaCBA piliation was shown to contribute to the growth of *L. rhamnosus* GG biofilm colonies, which appears to include a strong reliance on the adhesive SpaC tip pilin as cells aggregate together [14]. In this particular situation, the SpaCBA pilus is likely to be an adaptive advantage to *L. rhamnosus* GG and by its characteristic adhesiveness will help make this strain a less stringent allochthonous gut inhabitant [112].

The adhesive role of the SpaCBA pilus as a colonization factor is further demonstrated by its capacity to compete for binding sites in the gut mucosal epithelium. For instance, a recent study by Tytgat and colleagues had examined whether SpaCBA-piliated *L. rhamnosus* GG cells can supplant the binding of a vancomycin-resistant enterococcal species to mucus [113]. Here, the Gram-positive *E. faecium* E1165 strain is known to display sortase-dependent pili, which also happen to be quite homologous to the SpaCBA pili, reaching upwards to at least 50% sequence identity. By using polyclonal antibodies with specificity toward *L. rhamnosus* GG cells and the SpaC pilin, the authors were able to show that these immune sera can obstruct the mucus-binding ability of *E. faecium*, which presumably is due to its own surface pili. This binding interference was further linked to *L. rhamnosus* GG SpaCBA piliation by the observation that purified SpaC protein can also prevent *E. faecium* cells from adhering to mucus. As inferred from these findings, the authors suggested that the apparent outcompeting effect exerted by the SpaCBA pilus exemplifies some of the mechanistic rationale behind the advocated probiosis of the *L. rhamnosus* GG strain.

### 4.4. Immunogenicity

Although the published reports describing *L. rhamnosus* GG as a piliated strain were on hand during 2009 [11,13], it was only until three years later when a study came out that provided some first insights about the immunomodulating properties of the SpaCBA pilus [106]. Here, Lebeer and colleagues examined the phenotypic variances of different *L. rhamnosus* GG knockout mutants, including of the *spaCBA* operon, and found that the SpaCBA pilus is able to dampen the lipoteichoic acid (LTA)-induced mRNA levels of the inflammatory cytokine interleukin (IL)-8, for which it was suggested the related interactive mechanism might also involve the activation of the innate Toll-like receptor-2 (TLR2). Further to this, the authors of another study had concluded that, on the basis of a comparison between *L. rhamnosus* GG and two non-piliated strains of *L. casei*, the SpaCBA pilus should likely participate in TLR2-dependent NF-κB signaling [97]. Lending further support to the above findings, the added evidence from the study by von Ossowski and colleagues had suggested the SpaCBA pilus plays, in all likelihood, some sort of contributory role as a TLR2 agonist or activator [104]. Here, it was established that recombinant SpaCBA-piliated lactococci could activate NF-κB responses in HEK-TLR2 cells, which in itself was taken as an indication of stimulated TLR2-dependent activity. However, by using mutated lactococcal constructs, in which the SpaB and SpaC subunits had been removed from recombinantly expressed SpaCBA pili, it was shown that neither of these two ancillary pilins is specifically recognized by TLR2 [104]. Instead, it was reasoned that TLR2 recognition of SpaCBA pili might have more to do with the topology and overall protein fold of the polymerized pilin subunits [104]. This line of logic was consistent with the findings of another study, wherein the RrgA tip-pilin of the *S. pneumoniae* type 1 pilus could only activate TLR2-dependent responses while as an aggregated macromolecular form, and not in its monomeric state [83]. Yet, beyond its activation of TLR2-related signaling, the von Ossowski et al. (2013) study had revealed that the SpaCBA pilus is also involved with modulating the dendritic cell (DC) production of various inflammatory cytokines, such as IL-6, IL-10, and IL-12, and as well tumor necrosis factor-alpha (TNF-α). Noticeably, though, what emerged from this work was to be a recurrent theme involving the adhesive and surface-localized nature of SpaCBA piliation. Because innate immune signals have a short half-life, they require being released nearby to function effectively. For this, the SpaCBA pilus and, in particular, its SpaC tip-pilin adhesin would seem to provide the needed cell-to-cell contact, such that the closer proximity between immune-related cells and bacteria allows the pili (or perhaps some other types of surface features) to then promote the release of, e.g., TLR2-dependent responses or DC inflammatory cytokines [104]. Additionally (and going out on a speculative limb), it was suggested that the SpaCBA pilus might represent a novel microbe-associated molecular pattern (MAMP)-like modulator of innate immunity, and thus, in the case of *L. rhamnosus* GG, it would be one of several other cell surface structures that have been branded as immunomodulatory [104].

Further highlighting the immuno-adhesiveness of the SpaCBA pilus were the results from several studies examining other immunogenic responses and signaling pathways from a mix of immune-related cells. For instance, Ardita and colleagues showed through the use of a *spaC*-negative isogenic *L. rhamnosus* GG mutant that the SpaC subunit has a discernible role in stimulating certain types of cellular responses from human intestinal epithelial cell lines and the murine small intestine [107]. Here in this study, these included stimulating reactive oxygen species (ROS) production, pathway activation involving extracellular signal-regulated kinase (ERK) and mitogen-activated protein kinase (MAPK), and protection from intestinal damage induced by radiation. In the work by Ganguli et al. (2015), the focus was on human fetal gut epithelial cells, and where purified recombinant *L. rhamnosus* GG SpaC pilin was shown to cause a reduction in IL-6 levels when exposed to IL-1β, but as well, a modulation of immune gene expression by down-regulating TLR3, TLR4, and TIRAP (a TLR-associated intracellular adapter molecule) mRNA levels, yet up-regulating the levels for IL-1RA (IL-1 receptor antagonist) mRNA [114]. On the other hand, it was seen from the findings reported by Vargas García and colleagues that the SpaCBA pilus was also involved with modulating anti-inflammatory cytokine mRNA levels in macrophages, in this case by up-regulating IL-10 and down-regulating IL-6 in the RAW 264.7 murine cell line [115]. However, SpaCBA piliation was itself found not directly responsible for these immunomodulatory effects, but more likely the means used for maintaining contact between macrophage cells and other *L. rhamnosus* GG cell-surface attributes. Interestingly, an entirely different premise for the immunomodulating behavior of the SpaCBA pilus was proposed in the study by Tytgat and colleagues [116]. Here, the authors had identified the presence of mannose and fucose moieties on *L. rhamnosus* GG SpaCBA pili, presumably in the form of glycosylation, which were shown to be recognized by primary DCs through an interaction with DC-SIGN (Dendritic Cell-Specific Intercellular adhesion molecule-3-Grabbing Non-integrin), a C-type lectin receptor found on the surface of these cells and macrophages. Significantly, in addition to functioning as an adhesin, DC-SIGN is also known to initiate innate immune responses via TLR-dependent mechanisms. Related to this, Tytgat and colleagues had shown the elevated gene expression for certain DC cytokines (IL-6, IL-10, IL-12A, and IL-12B) that was roused by the mannosylated/fucosylated SpaCBA pili could be partly reduced by the blocking action of DC-SIGN antibody [116].

However, in spite of its immuno-characteristics, the results reported on the *L. rhamnosus* GG SpaCBA pilus should be interpreted in a broader context. For instance, the magnitude of innate responses from the host gut immune system toward commensals (and probiotics) is much more subdued than to any opportunistic pathogens, and then what can be best inferred as only basal-level immune activities between various MAMPs present on the outer bacterial surface and the pattern recognition receptors (e.g., TLRs) of host-immune cells [117,118,119]. Despite this, over the past few years considerable research effort has been geared at understanding the immunogenic basis for the advocated health benefits of certain gut-residing (and transient) commensals, for which much of the focus is on a variety of outer surface features, both proteinaceous and carbohydrous. Regarding the SpaCBA pilus, its own recognition by the host innate immune defenses is to be expected, but while the various response strengths are more or less at low stimulatory levels, this would be consistent with the borderline immuno-intrusiveness of *L. rhamnosus* GG as a harmless gut-adapted commensal. Still, as an envisaged benefit to the host, one can theorize that this commensal-derived pilus is possibly correlated with sensitizing the intestinal epithelium to an immune-alerted readiness state, which itself could conceivably be one of the protective mechanisms that specifically thwarts gut-invading piliated pathogens.

## 5. SpaFED Piliation

The first published account of the *spaFED* operon, which encodes for the second type of lactobacillar pilus called SpaFED, comes from the study by Kankainen and colleagues, wherein the related pilin (*spaF*, *spaE*, and *spaD*) and sortase (*srtC2*) genes are found in the genomes of the *L. rhamnosus* GG and LC705 strains [11]. Surprisingly, since this original finding, along with the observed incidence of the fimbrial *spaFED* operon in additional *L. rhamnosus* strains [10,12] and in two other *Lactobacillus* species [92,93,94], there have still been no reports of a natively produced SpaFED pilus structure. Thus, by comparison to the published studies on SpaCBA piliation, the extent of characterization available for the SpaFED pilus is less comprehensive, with most findings about its functional and structural properties relating to a recombinantly expressed form in *Lactococcus lactis* [98]. However, just as a caveat, beyond the phenotypic revelations about the SpaFED pilus, any applicability in vivo in the original host species or strain is quite notional and should be viewed with some circumspect. 

### 5.1. Genetics

While the fimbrial *spaFED* operon (i.e., *spaF*-*spaE*-*spaD*-*srtC2*) can only be detected in the genomes of the three *Lactobacillus* species from the casei group, i.e., *L. casei*, *L. paracasei*, and *L. rhamnosus* [92,93,94], it appears to be a nearly common feature within all the related strains. Moreover, shared among these species and strains is an absence of experimental evidence for any active gene expression from the *spaFED* operon, which in effect makes the SpaFED pilus a conjectural structure with no genuine role to play in these lactobacilli. Thus, in contrast to those *spaCBA* operons in a few *L. rhamnosus* strains that can be constitutively expressed, such as through an upstream activating *iso*-IS*30* element [97], the *spaFED* genes lack this capacity and instead lay dormant. On the other hand, whether the expression of the *spaFED* operon is inducibly controlled via some sort of stimulus-responsive promoter is not yet certain [12]. For instance, although varying the conditions of growth (e.g., nutrients and temperature) did not trigger the activation of the *spaFED* operon in *L. rhamnosus* GG [90], gene expression might still be induced by other environmental stimuli, or then *in situ* by the intestine as reported for the Tad (tight adherence) pili of the *Bifidobacterium breve* UCC2003 [120].

Nonetheless, in the Rintahaka et al. (2014) study, the near-upstream DNA region of the *spaFED* operon from 13 different *L. rhamnosus* strains was aligned and examined in silico for the presence of any canonical regulatory elements [98]. For this, sequence found directly upstream of the coding region for the *spaF* gene had corresponded to a ribosomal binding site (RBS), which itself was also identified for the *spaE*, *spaD*, and *srtC2* genes. As it was also found that encoded for each of these pilus genes is an open reading frame without any premature stop codons, the *spaFED* operon appears to be a complete translational unit, and thus at this level there is no reason preventing a native (or recombinant) pilus from ultimately being assembled together. Added scrutiny of DNA sequence further upstream had not identified a symmetric operator site for binding a repressor protein, which seemed to rule out inducible gene expression requiring this type of regulatory element [98]. However, while this study did locate the consensus sequence for the -10 and -35 promoter recognition sites, along with possible nucleotides for a transcriptional start site, additional sequence elements for either inducible or constitutive gene expression were not evident. This upstream region had also lacked the DNA elements associated with the attenuation mechanism for controlling *S. gallolyticus* pilus expression [35]. Moreover, since a stem-loop DNA motif for a possible transcriptional terminator was not identified anywhere between the -10 and -35 promoter region and the start of the *spaF* gene, a non-expressible *spaFED* operon is less likely to occur for this reason, but which alternatively might be explained by regulatory sequence that has become somehow corrupted.

Although results from a pan-genome study of *L. rhamnosus* [12], and similarly of *L. casei* and *L. paracasei* [92,93], suggest that the *spaFED*-related genes are a commonality in various strains and part of the core genome, it remains puzzling why the *spaFED* operon would still persist in the genome, particularly as without any pilus gene products this would not offer cells an adaptive advantage or fitness benefit. Yet, as it seems carrying the *spaFED* operon does not impose an additional genetic load or liability on the casei group of lactobacilli, the corresponding genes have resisted the evolutionary forces of loss or decay, and thus continue to remain a constant genomic attribute, though for what cellular purpose or specific function is uncertain.

### 5.2. Structure

The undocumented status of the SpaFED pilus in the *L. rhamnosus*, *L. casei*, and *L. paracasei* species has precluded the structural characterization of its native form. However, as the *spaFED* operon was predicted to be an intact translational unit [98], it was undertaken to clone and express the *spaFED*-related genes of *L. rhamnosus* GG in the recombinant host of *L. lactis*. Based on results from electrophoretic immunoblotting and immuno-EM, it was found that the *spaF*, *spaE*, *spaD*, and *srtC2* genes of the *spaFED* operon were readily transcribed and translated recombinantly in lactococcal cells to produce a surface-assembled pilus [98]. From single and double immunogold-labeling experiments, it was revealed that the *spaFED* operon encodes a prototypical sortase-dependent pilus, for which the backbone SpaD (~51 kDa), basal SpaE (~45 kDa), and tip SpaF (~104 kDa) pilin subunits are structurally located in their expected sites. However, while it appeared that the ancillary SpaF and SpaE pilins are also present along the length of the SpaFED pilus backbone, they were interpreted to be a gold-particle immunolabeling artifact [98].

To date, there are no reports in the literature that any of the SpaFED pilins has had its tertiary structure solved by X-ray crystallographic methods. However, there are two published studies describing the successful crystallization of the backbone SpaD [121] and basal SpaE [122] subunits, along with their preliminary X-ray diffraction analysis. For SpaD, sodium iodide-soaked orthorhombic crystals yielded diffraction data to a resolution of 2.2 Å from which an interpretable electron-density map was generated by single-wavelength anomalous diffraction (SAD) phasing [121]. For SpaE and its SAD phasing, selenium-substituted orthorhombic crystals were able to diffract to 1.8 Å, with a reasonably strong anomalous signal observed in the X-ray data [122]. Correspondingly, model building and refinement are said to be in progress for both types of pilin structures.

### 5.3. Adhesion

Aside from the presence of domains for the CnaA or CnaB Ig-like fold as a structural stalk, none of the primary structures for the *L. rhamnosus* SpaFED pilins are predicted to have homology to a familiar binding domain [11]. However, based on its molecular size and thus likely location at the pilus tip, the SpaF pilin would otherwise be expected to have binding properties. This in fact was established in the study by von Ossowski et al. (2010), with the finding that recombinant SpaF protein is able to adhere to intestinal mucus and at a level similar to that of SpaC pilin [102]. Moreover, as neither basal SpaE nor backbone SpaD was shown to have the same adhesion capacity, it would appear that the SpaF subunit is solely responsible for the mucus-binding property of the SpaFED pilus. This latter point was subsequently confirmed in the Rintahaka et al. (2014) study through the use of lactococcal clones expressing WT and SpaF-deleted SpaFED pili [98]. Here, it was revealed that the lactococcal cells with WT SpaFED pili had bound readily to mucus, which was related to the SpaF pilin since the lactococci having SpaF-deleted pili could no longer display a similar level of substrate specificity. Moreover, by using the same two SpaFED-piliated lactococcal clones, it was also shown that the SpaFED pilus can adhere to ECM proteins (i.e., fibronectin and collagens I and IV) and human intestinal Caco-2 and HT-29 epithelial cells, where in all cases the main determinant of binding was judged to be the SpaF tip pilin. Surprisingly though, in another study [14], it was found that the binding capacity of the SpaFED pilus did not lend itself to helping form biofilm, as this ability was not detectable with the SpaFED-piliated lactococci. Apart from this latter finding, in terms of adhesive interactions with mucus, ECM proteins, and gut epithelial cells, the SpaFED pilus can still be seen as functionally akin to its SpaCBA counterpart. 

### 5.4. Immunogenicity

Again, through the use of WT and SpaF-deleted SpaFED-piliated lactococcal clones [98], SpaFED piliation was examined for any innate immunogenic properties, much like was done previously for the SpaCBA pilus [104]. For the immuno-characterization of the SpaFED pilus, it was examined whether the two SpaFED-piliated lactococcal constructs can prompt a TLR2 response, which was inferred from the measured NF-κB activation in HEK-TLR2 cells. In addition, these SpaFED-piliated lactococci were tested for their effect on the endogenic levels of IL-8 in Caco-2 cells. Surprisingly, the WT SpaFED pilus did not produce the same stimulatory effects as the SpaCBA pilus or as the bifidobacterial [15] and streptococcal [83] sortase-dependent pili, but rather instead it behaved in an opposite manner by dampening the levels of TLR2-induced activation and IL-8 production [98]. Somewhat intriguingly, this dampening influence was not observed with the SpaF-deleted pilus, which seemed to suggest the SpaF subunit might have an adhesive-driven role in lowering the tested immune responses [98]. In the speculative scenario of the gut, a natively produced SpaFED pilus would thus offer an opposing innate immunogenic role than that of the SpaCBA pilus, but granted if these two pilus types were to be expressed at the same time, e.g., by *L. rhamnosus* GG cells, it is conceivable that they could contribute to a state of immune tolerance by providing a localized counterbalance to the induced high-and-low immuno-responsiveness of the host [98].

## 6. LrpCBA Piliation

The third type of sortase-dependent pilus in lactobacilli is associated with the *L. ruminis* species and called LrpCBA, where its prefix name is derived from “*L*. *ruminis* pilus” [14]. In comparative terms, *L. ruminis* as a surface-piliated host is quite unlike the *L. rhamnosus* species since it is one of the few lactobacilli that is motile by flagella [95,123,124,125], strictly anaerobic [126], and confined to only the gut environment [124], where in fact its colonization of this ecological niche is considered autochthonous (indigenous) [127,128]. Although the related pilus loci had been identified in the *L. ruminis* genome as early as 2009 (unpublished observations; see [14]) and then later in 2011 in the published work by Forde et al. (2011) [95], it was only until 2015 when Yu and colleagues produced results confirming that LrpCBA pilus structures were actively produced by this lactobacillar species [14]. Thus far, the majority of work done to characterize the LrpCBA pilus comes from this particular study on *L. ruminis* and, for this reason, the various properties determined for it are limited and not as far-reaching as for SpaCBA piliation. 

### 6.1. Genetics

Genetically, the LrpCBA pilus follows suit with other sortase-dependent pili by having its genes clustered into an operon [14,129]. Here, the loci for the predicted tip (LrpC), basal (LrpB), and backbone (LrpA) pilins and C-type sortase (SrtC) are arranged in tandem on the *L. ruminis* genome as the fimbrial *lrpCBA* operon, i.e., *lrpC*-*lrpB*-*lrpA*-*srtC* [14,129]. Almost invariably, every strain of *L. ruminis* that has been examined so far is containing the *lrpCBA* loci, and based on a pan-genome survey of strains from various host sources (human, bovine, porcine, and equine) all of them but the *lrpB* gene are found to be part of the core genome. Of note, *lrpB* was excluded from the core genome because of certain nucleotide inconsistencies in the bovine- and equine-sourced versions that caused a reading-frameshift change to be present [129]. However, as it remains uncertain if these represent an authentic indel mutation or a DNA sequencing error, *lrpB* might itself be put among the *L*. *ruminis* core genes [129].

Results from the Yu et al. (2015) study [14] indicated that the primary structures of the *lrpC*, *lrpB*, and *lrpA* genes from the human-derived *L*. *ruminis* ATCC 25644 strain exhibit the typical sequence characteristics of a Gram-positive pilin subunit, such as an E-box and pilin motif, along with the established domains for both N-terminal secretion signaling and C-terminal sortase recognition (Table 1). Further, while an amino acid sequence alignment of the predicted *lrpCBA*-encoded proteins from human, bovine, porcine, and equine isolates of *L*. *ruminis* showed a high level of shared identity (e.g., ranging from 89.7% to 100%), this was unobserved with alignments of the *L*. *rhamnosus* GG *spaCBA-* and *spaFED*-encoded pilus proteins [14]. This finding lends support to LrpCBA piliation being a third lactobacillar pilus type. Of added interest, the *lrpCBA* operon appears to be genome-specific to only *L*. *ruminis*, as a BlastP search of the NCBI database using the LrpCBA pilin-proteins revealed no counterparts in any other *Lactobacillus* species [14]. This observation contrasts with that of the *spaCBA* and *spaFED* operons, both of which can be found in the genomes of more than one species, i.e., *L*. *rhamnosus*, *L*. *casei*, and *L*. *paracasei* [12,92,93,94].

As the microarray analysis of *L. ruminis* cells had already indicated the *lrpCBA* genes are actively transcribed, for which their expression is more up-regulated in a human-isolated strain than one originating from a bovine source [95], this was followed up by a study that examined the DNA region that precedes the *lrpC* gene for the presence of any possible regulatory elements [14]. Based on an aligned 106-bp DNA segment from the genomes of various host strains of *L. ruminis* (human, bovine, porcine, and equine), a putative RBS motif of 5′-GGAGAG-3′ and a pair of hexanucleotide sequences resembling the -10 and -35 consensus promoter regions were identified [14]. In addition, a candidate purine nucleotide was proposed as a possible starting point for transcribing the genes of the *lrpCBA* operon [14]. Based on these findings it was reasonably concluded that such sequence elements are what would presumably regulate constitutive *lrpCBA* gene expression in *L. ruminis*.

### 6.2. Structure

Given that active expression of the *lrpCBA* genes had been reported to occur in *L. ruminis* cells [95], there was all likelihood that the corresponding transcripts would be translatable into the specific protein products needed for building up the LrpCBA pilus. This in fact was the outcome as the constitutive production of “native” LrpCBA pili was observed on the cell surface of *L. ruminis* [14] (Figure 3). Convincing evidence of the LrpCBA pilus as a fully assembled surface-anchored structure was initially demonstrated for the human-isolated ATCC 25644 strain [14] and subsequently for the porcine GRL1172 strain [130] by using the customary immunoblotting and immuno-EM approaches employed for the same purpose with the SpaCBA and SpaFED pili [11,90,98]. Here, through single and double immunogold-labeling of pilin-proteins, EM had helped visualize LrpCBA piliation in the ATCC 25644 strain as being typical of other sortase-dependent pili, with the tip LrpC (~123 kDa), basal LrpB (~39 kDa), and backbone LrpA (~49 kDa) pilins assuming their predicted roles in the assembled structure [14]. Once again, though, the immuno-labeling artifact of ancillary pilins (LrpC and LrpB) along the pilus backbone seemed apparent. Additionally, the electron microscopic examination of the overall *L*. *ruminis* cell population revealed that the outer surface of most cells is piliated, with an average of one to five pili per bacterium [14], which is on the order of ten-fold less than estimated for *L. rhamnosus* GG SpaCBA piliation [11]. Further, by comparison, the EM-deduced structure of the recombinantly produced LrpCBA pilus in *L. lactis* is remarkably authentic looking, notwithstanding its tendency to be somewhat lengthier than the native version [14]. 

When a subunit size comparison is made between the various types of LrpCBA pilins and their counterparts in the SpaCBA and SpaFED pili, LrpC, SpaE, and SpaD are the largest (123, 45, and 51 kDa, respectively), whereas SpaC, SpaB, and SpaA are the smallest (90, 20, and 30 kDa, respectively) [11,14,90,98]. Put in the context of the polymerized backbone pilins, the SpaCBA pilus can be projected as retaining the narrowest structural width, with the LrpCBA and SpaFED pili both then being wider and of somewhat similar thickness. Thus, at the molecular level, for the two pilus types that are known to be natively produced by cells of *L. ruminis* and *L. rhamnosus*, one might interpret their potential difference in backbone girth (i.e., inferred from the subunit sizes 49 kDa for LrpA and 30 kDa for SpaA) most likely points to the LrpCBA pilus having the structural characteristics and ability to better tolerate the environmental stress of the intestinal habitat, e.g., by being thicker and stronger, and thus less susceptible to breakage or shearing at longer lengths. Consequently, it is more than tempting to speculate that from the autochthonous perspective of host colonization, this particular aspect of the LrpCBA pilus structure can confer an adaptive advantage or gain to gut-dwelling *L. ruminis* cells.

### 6.3. Adhesion

Among the three types of LrpCBA pilins, only the LrpC primary structure is homologous to a protein domain that is predicted to be substrate binding. Here, the tip LrpC pilin has homology to a collagen-binding domain, and one which differs from those CnaA or CnaB domains that serve as a stalk-like component of most Gram-positive pilins [14]. This predicted homology contrasts with the *L. rhamnosus* SpaC and SpaF tip pilins, as neither shares any sequence similarity with collagen-binding proteins [11,112], albeit that both were ultimately shown to adhere to collagen substrates [98,108]. For the LrpCBA pilus, its affinity for collagen protein was demonstrated by using recombinant lactococcal clones that produce WT and LrpC-deleted LrpCBA pili [14]. Here, it was observed that the WT LrpCBA pilus can readily adhere to collagen, binding more to type I than type IV [14]. As adhesion to these collagen types is substantially diminished for the LrpC-deleted pili, this suggested the LrpC subunit has a requisite adhesive role in the binding of collagen by LrpCBA pili [14]. In the context of *L. ruminis* itself, this adhesion capacity of the LrpCBA pilus is in keeping with the collagen-binding ability found with the ATCC 25644 strain [14] and other host-sourced isolates [130]. As *L. ruminis* cells can also adhere to fibronectin [14,130], the ability to do so was partially attributed to the LrpCBA pilus, in which LrpC acts as the main source of binding [14], and in spite of its lack of homology to any other fibronectin-binding proteins [14,129]. Likewise, a similar trend was observed for the binding of the LrpCBA pilus to human Caco-2 and HT-29 gut epithelial cells [14]. However, unlike found for the SpaCBA and SpaFED piliation [11,98,112], the LrpCBA pilus lacks the ability to be mucoadhesive [14]. Although this nonbinding ability toward mucus is a clear departure from the other lactobacillar pili, it remains consistent with the fact that there are no encoded proteins in the *L. ruminis* genome that display any similarity to proven mucus-binding domains [129] and that *L. ruminis* (e.g., the ATCC 25644 strain) is itself an extremely poor binder of mucus [14]. Interestingly, the functionality of LrpCBA piliation also departs from that of the SpaCBA pilus [14,106] and other types of Gram-positive pili [131,132] as evidence suggests it has no adhesive role in the self-aggregation of cells to produce biofilm, even though *L. ruminis* demonstrates a strong biofilm-forming phenotype [14]. Thus it seems that while the formation into biofilm communities by some piliated bacteria is seen as adaptively advantageous during niche development and expansion, for *L. ruminis* cells their LrpCBA piliation would have no particular contribution to such ecological processes.

Then again, for the ecological aspects underlying the gut autochthony of the *L. ruminis* species, the bi-functional binding specificity of the LrpCBA pilus toward collagen and fibronectin proteins represents an effective colonization strategy for bacterial attachment to the intestinal epithelium, and overtly to any exposed location along the ECM layer (Figure 4). Together with its other characteristics (e.g., motility, poor mucoadhesiveness, and strict anaerobiosis), LrpCBA-piliated *L. ruminis* would be well-equipped to avoid and get past the mucosal barrier and then establish a stable cell population in the specific micro-environment of folded and deoxygenated crevices found throughout the epithelium lining of the gut. Undoubtedly, the first-contact ability of an ECM-adhesive LrpCBA pilus can contribute to helping promote and support the indigenous lifestyle of *L. ruminis* in the GI tract.

### 6.4. Immunogenicity

While there are some published accounts characterizing the innate immunogenic potential of *L. ruminis* cells [14,130,133], only one study thus far has specifically scrutinized the related role played by LrpCBA piliation [14]. This investigation was performed similarly to what had been done for the *L. rhamnosus* SpaCBA and SpaFED pili [98,104] and involved the use of recombinant LrpCBA-piliated *Lactococcus* constructs, one WT and another LrpC-deleted. Interestingly, although live cells of the *L. ruminis* ATCC 25644 strain were able to potentiate increased TLR2-dependent NF-κB signaling and endogenic production of IL-8 in the recombinant HEK-TLR2 cell line, the WT LrpCBA pilus aroused the opposite effect and had a dampening influence instead [14]. A similar result occurred for the endogenous IL-8 secretion in human intestinal Caco-2 cells, as some moderate lowering of the levels was observed with WT LrpCBA pili [14]. However, whereas these findings were in accordance with the immuno-dampening exerted by the SpaFED pilus [98], there was no causal role for the tip LrpC subunit since its removal from the LrpCBA pilus had no counter influence. This contrasts with the tip SpaC and SpaF pilins of the SpaCBA and SpaFED pili, respectively, as their presence and intrinsic adhesiveness appeared necessary for any immunomodulatory activities [98,104]. Seemingly, as the LrpC constituent of the LrpCBA pilus bears no direct responsibility for the dampened immune responses from intestinal or immune-related cells, it was suggested this diminishing effect could be based on the recognition of the overall topological fold for the pilus structure [14]. On the other hand, although there appears to be an LrpCBA pilus-mediated dampening of IL-8 levels, another study reported that the interaction between TLR5 and the flagella of *L. ruminis* stimulates increased IL-8 secretion from Caco-2 cells [123]. In practice, this would suggest that via the immunostimulatory capacity of their pili and flagella, *L. ruminis* cells are able to simultaneously fluctuate the production of IL-8 from host intestinal cells, which in itself might represent a type of homeostatic counterbalancing mechanism that allows for host immune tolerance and, in doing so, an autochthonous presence in the gut [14].

## 7. Conclusions

Irrespective of its Gram-positive host, the sortase-dependent pilus can be viewed as a molecular engineering coup for those bacteria whose outer surfaces display this particularly long and adhesive protrusion. Undoubtedly, the building-block simplicity of piecing together various role-defined pilin subunits represents a successful genetic and evolutionary adaptive strategy for such bacteria to gain their initial foothold in a biological niche or habitat. For the three types of lactobacillar piliation presented in this review (i.e., SpaCBA, SpaFED and LrpCBA), each has been described as having the basic characteristics common to all sortase-dependent pili, but as well, certain unique properties and associated actions that are inherent to them. As for this latter point, it is likely these pilus types exhibit some of the phenotypic and physiological variation that conveys an ecological advantage to their respective bacterial hosts. However, still remaining unresolved is what native role might be played by the SpaFED piliation of the casei group of lactobacilli as well as what additional number of *Lactobacillus* species predicted as containing sortase-dependent pilus genes are in fact surface-piliated. In all likelihood, by tackling and solving these challenges this will open up new chapters of scientific discovery to the continuing and unfolding story of sortase-dependent pili in Gram-positive commensal bacteria.

## Figures and Tables

**Table 1:** Overview of conserved amino acid sequence motifs and domains in the lactobacillar SpaCBA, SpaFED, and LrpCBA pilin subunits. *

Primary Structure Feature	Tip Pilin Subunit
N-terminal secretory domain	MTAKVARTGHLFAVLLILMSMLTGLVTSGSSVVT (SpaC) LPRKWIHMLMLLLMLVTQIGSA (SpaF) MERNKIFKKLLCILGAVATVFAIVFAMGKFDGEKANA (LrpC)
C-terminal LPXTG domain	LPHTGGQGYQRLLGIALGLISAAFLLLLVVLIKRRVVKQHD (SpaC) LPKTGGSGILLFLMVAISACGGGWLLYLYLKRKEAR (SpaF) LPQTGGPGRLLFEALGSLLIVVACALTEVLIWRRIRSSKGV (LrpC)
Pilin motif	not detected (SpaC) not detected (SpaF) not detected (LrpC)
E-box	YGIQEAAAPTGY and YTMSETKAPDGY (SpaC) YRLTETKAPAGF (SpaF) YKLVETRTQSGY (LrpC)
**Primary Structure Feature**	**Basal Pilin Subunit**
N-terminal secretory domain	MTKSFRPLVILTFCLALLVSLATTTLQQTQA (SpaB) MRRFYWWLVPLLLLIGIVLGNTPHWVHA (SpaE) MKRVLKLLFMIVAFMTAVFAGSGQASA (LrpB)
C-terminal LPXTG domain	LPQTGDTVAAWLSVLGLIIFATVLAFNIKNQKINKWER (SpaB) LPAMSDWRNLRFVLLGSLLLLLATYFFIKNKKARHHACK (SpaE) LPQTGEAKSIMALLGIGIICLVVLVSVGRRNYKEEH (LrpB)
Pilin motif	TADFWQLVSKN (SpaB) PLQTIHLYPKN (SpaE) FPLGGQSYAKN (LrpB)
E-box	YLFKETAAPKNI (SpaB) YFFEELQGVPGY (SpaE) YYFSEVQAPKGY (LrpB)
**Primary Structure Feature**	**Backbone Pilin Subunit**
N-terminal secretory domain	MKKTIAKKVLTLTSTILMTLLMVLGFNGTRVQA (SpaA) MQVTFKKIGHSLLAALMLMSFLLPLLSAGKPVHA (SpaD) MKNHKKLRNALATLLLALPLALQGAVGVKTAQA (LrpA)
C-terminal LPXTG domain	LPHTGGTGTVIFAILGVALIAFGAVAYRKRRNGF (SpaA) LPMTGGIGLFAFLMIGAILMGGGHLMKKKTSKKV (SpaD) LPSTGGMGIVLFIAAGVVVMAGAAGTMIVRRNRRENI (LrpA)
Pilin motif	ADGNVYVYPKN (SpaA) DLTNIHLYPKD (SpaD) VQKSINIYPKN (LrpA)
E-box	YLFHETNPRAGY and YTAVETNVPDGY (SpaA) YAFHEAVTPQPY and YTLVETAAPEGY (SpaD) YLFAETDAPANI and YAVKEVKAPTGY (LrpA)

* Sequence motifs and domains for the pilin subunits are based on the deduced primary structure from the pilus operon genes in the *L. rhamnosus* GG (*spaCBA* and *spaFED*) and *L. ruminis* ATCC 25644 (*lrpCBA*) strains.
